# Spectroscopic characterization of a thermodynamically stable doubly charged diatomic molecule: MgAr^2+^

**DOI:** 10.1039/d1cp00730k

**Published:** 2021-04-30

**Authors:** Dominik Wehrli, Matthieu Génévriez, Frédéric Merkt

**Affiliations:** Laboratory of Physical Chemistry, ETH Zurich CH-8093 Zurich Switzerland merkt@phys.chem.ethz.ch

## Abstract

Although numerous doubly positively charged diatomic molecules (diatomic dications) are known from investigations using mass spectrometry and *ab initio* quantum chemistry, only three of them, NO^2+^, N_2_^2+^ and DCl^2+^, have been studied using rotationally resolved optical spectroscopy and only about a dozen by vibrationally resolved double-ionization methods. So far, no thermodynamically stable diatomic dication has been characterized spectroscopically, primarily because of experimental difficulties associated with their synthesis in sufficient densities in the gas phase. Indeed, such molecules typically involve, as constituents, rare-gas, halogen, chalcogen, and metal atoms. We report here on a new approach to characterize molecular dications based on high-resolution photoelectron spectroscopy of the singly charged parent molecular cation and present the first spectroscopic characterization of a thermodynamically stable diatomic dication, MgAr^2+^. From the fully resolved vibrational and partially resolved rotational structures of the photoelectron spectra of ^24^MgAr^+^ and ^26^MgAr^+^, we determined the potential-energy function of the electronic ground state of MgAr^2+^, its dissociation (binding) energy (*D*_0_ = 10 690(3) cm^−1^), and its harmonic (*ω*_e_(^24^MgAr^2+^) = 327.02(11) cm^−1^) and anharmonic (*ω*_e_*x*_e_(^24^MgAr^2+^) = 2.477(15) cm^−1^) vibrational constants. The analysis enables us to explain quantitatively how the strong bond arises in this dication despite the fact that Ar and Mg^2+^ both have a full-shell rare-gas electronic configuration.

## Abstract 2

We present a new method to study doubly charged molecules relying on high-resolution spectroscopy of the singly charged parent cation, and report on the first spectroscopic characterization of a thermodynamically stable diatomic dication, MgAr^2+^.

## Graphical abstract

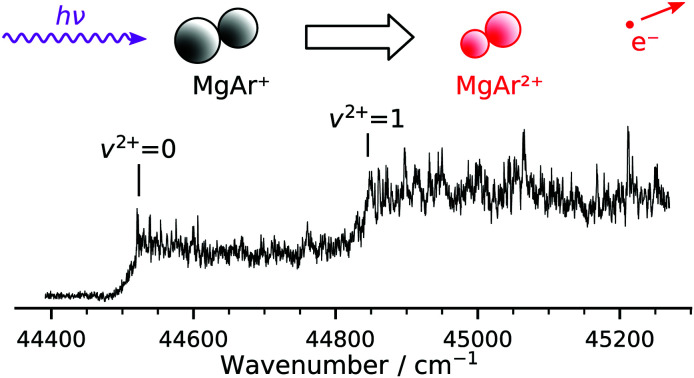

## 1 Introduction

**Fig. 1:**
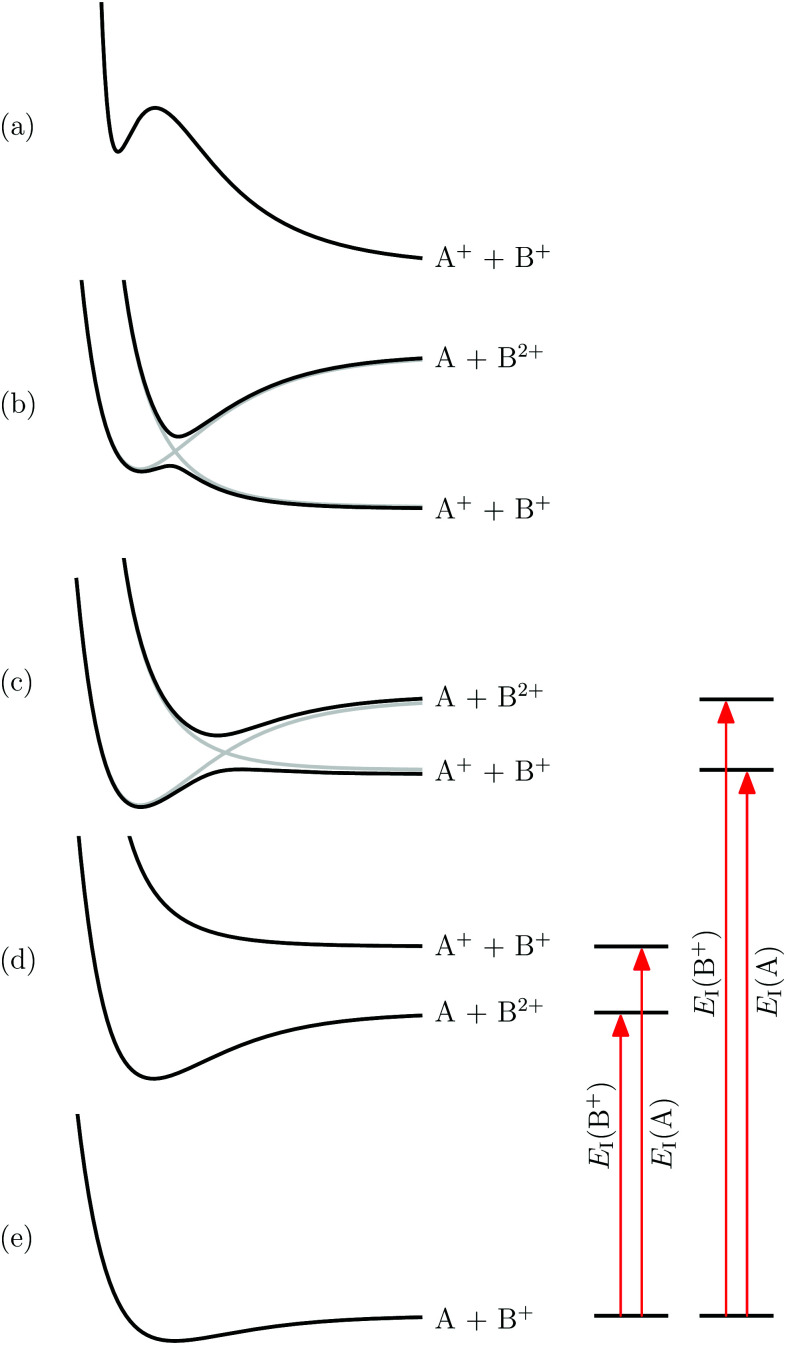
Schematic potential-energy functions for molecules of the type BA^2+^ corresponding to the situations leading to metastable DIDIs (a and b) and thermodynamically stable DIDIs (c and d). (e) Potential-energy function of the singly charged precursor molecule BA^+^. Right of panels (c–e): arrows indicating the energetic order of the dissociation limits dependent on the ionization energies of A and B^+^. Inspired by Fig. 2 of ref. 4.

Doubly positively charged diatomic molecules BA^2+^, called diatomic dications (DIDIs) hereafter, are intriguing and elusive chemical species. They are encountered in plasmas and play a role in planetary ionospheres^1^ and more generally in molecular astrophysics.^2^ Their structure and reactivity are at the focus of numerous studies.^3–12^ The qualitative aspects of their stability are well known. The condition for their ground state to be thermodynamically stable is that the dissociation limit associated with the products A + B^2+^ in their ground states lies energetically below (Fig. 1(d)) or only slightly above (Fig. 1(c)) the dissociation limit associated with the products A^+^ + B^+^.^4,12,13^ This condition requires, in turn, the ionization energy of B^+^ to be smaller, or only slightly larger, than that of A (see red arrows to the right of Fig. 1(c)–(e)), which is only met if A has an unusually high and B^+^ has an unusually low ionization energy, as is the case for alkaline-earth–halide and alkaline-earth–rare-gas DIDIs. Falcinelli *et al.* have listed most candidates of thermodynamically stable DIDIs and identified several of them using mass spectrometry.^14^

The large majority of the DIDIs known today are metastable, *i.e.*, their lowest level is located above the dissociation asymptote A^+^ + B^+^. Metastability typically arises when the ionization energy of B^+^ is significantly larger than that of A and results from an avoided crossing between the repulsive potential function dissociating into A^+^ + B^+^ and the attractive potential function correlating with A + B^2+^ (see Fig. 1(b)), or if a potential barrier is formed at short range in the otherwise repulsive Coulomb potential by binding valence interactions^13,15–17^ (Fig. 1(a)). The best and earliest known case of a DIDI, He_2_^2+^, belongs to the latter category.^15,18,19^ Today, the most reliable source of information on the structure and binding of DIDIs is *ab initio* quantum chemistry, see ref. 20 for a recent example concerning thermodynamically stable DIDIs.

Experimentally, the vast majority of DIDIs has been identified by mass-spectrometric methods, which enable their unambiguous detection but do not provide structural information. Only very few DIDIs have been characterized by spectroscopic methods which provide quantitative information on their structure. High-resolution, rotationally resolved spectra have been obtained for only three DIDIs, N_2_^2+^,^21–25^ NO^2+^,^26^ and DCl^2+^,^27^ and the vibrational structures of about 10 DIDIs have been studied by double-photoionization coincidence methods starting from the ground state of the neutral molecules (see ref. 11 and references therein). All cases for which spectroscopic information is available concern metastable DIDIs and, to our knowledge, no spectroscopic information has ever been obtained on a thermodynamically stable DIDI. One of the reasons for this absence of spectroscopic data is that these dications typically involve rare-gas, halogen, or chalcogen, and metal atoms. It is thus difficult to generate potential precursor neutral or singly-charged molecules BA and BA^+^ in the gas phase with sufficient densities. Another reason is that the double-ionization thresholds of diatomic molecules lie in the vacuum-ultraviolet (VUV) or soft X-ray ranges and are difficult to reach with table-top laboratory radiation sources.

We present here the first spectroscopic characterization of a thermodynamically stable DIDI, MgAr^2+^. To obtain high-resolution spectroscopic information on this dication, we have recorded the photoelectron spectrum of MgAr^+^ using the techniques of pulsed-field-ionization zero-kinetic-energy photoelectron (PFI-ZEKE-PE)^28,29^ and mass-analyzed threshold ionization (MATI) spectroscopy.^30^ Until this work, high-resolution photoelectron spectroscopy of molecular cations had been deemed impossible because of (i) the very limited density of ions that can be generated in the gas phase as a result of space-charge effects and (ii) the high ionization energies of cations. We demonstrate here that high-resolution photoelectron spectroscopy can be applied to samples of less than 1000 state-selected cations and that cations can be efficiently ionized despite their high ionization energies by resonant multiphoton excitation.

The photoelectron spectra we obtained were sufficiently resolved (∼2 cm^−1^) to obtain information on the rotational contours of the successive vibrational bands of the photoelectron spectrum of MgAr^+^ and to determine isotopic shifts in the spectra of ^24^MgAr^+^ and ^26^MgAr^+^. From these observations, we could extract an accurate potential-energy function for the electronic ground state of MgAr^2+^, prove experimentally that it is thermodynamically stable, and analyze the nature of the bond. We found the binding energy to be more than 1.3 eV (125 kJ mol^−1^), *i.e.*, comparable to a typical covalent bond, despite the fact that both constituents (Mg^2+^ and Ar) have full-shell rare-gas electron configurations.

MgAr^2+^ had been observed by mass spectrometry prior to our work.^31–35^ It appears as an undesirable species in inductively-coupled-plasma mass spectrometry and complicates the chemical analysis of S isotopes.^34,35^ From the analysis of the density of MgAr^2+^ at different plasma temperatures, Hattendorf *et al.* could estimate the ground-state dissociation energy to be in the range between 124 and 130 kJ mol^−1^.^34^ In addition, Gardner *et al.*^36^ have characterized the electronic and vibrational structure of MgAr^2+^ in high-level *ab initio* quantum-chemical calculations. These studies provided very useful and important reference data with which our new results are compared.

**Fig. 2:**
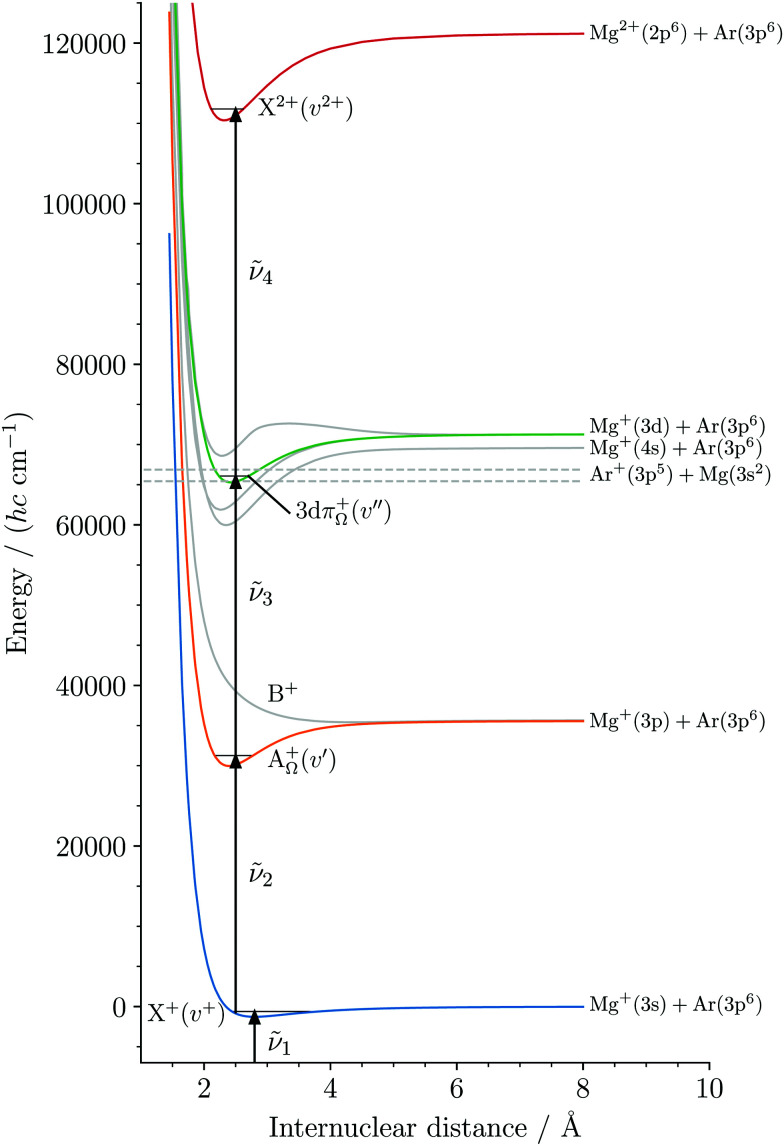
(1 + 1′ + 1′′) three-photon excitation sequence used to photoionize MgAr^+^ X^+^(*v*^+^) *via* the A_1/2_^+^(*v*′) and 3dπ_1/2_^+^(*v*′′) intermediate levels. MgAr^+^ was produced in its X^+^ ground electronic state by photoionization of metastable MgAr. The MgAr^+^ potential-energy functions were taken from ref. 39 and that of the ground state of MgAr^2+^ was taken from ref. 36.

Our approach to obtain spectroscopic information on DIDIs relies on the preparation of the precursor singly-charged molecule BA^+^ by photoionization of the neutral molecule BA. The photoelectron spectra of BA^+^ are then recorded following resonance-enhanced multiphoton excitation. The scheme used to study MgAr^2+^ is illustrated in Fig. 2, which depicts the potential-energy functions of all relevant states, as described in more detail in Section 2. It enables us to efficiently ionize MgAr^+^, despite its high ionization energy of about 13.9 eV, using commercial lasers operating in the UV region of the electromagnetic spectrum. This aspect is of central importance in the present work because of the low densities (typically ∼10^4^ cm^−3^) and low numbers (typically 200 per experimental cycle at a repetition rate of 25 Hz, *i.e.*, 5000 s^−1^) of MgAr^+^ ions generated in our experiments.

The term symbols and quantum numbers used to designate the different states are cumbersome. To define and simplify the notation of states and transitions, we use the nomenclature summarized in Table 1. For example, 3dπ_1/2_^+^(*v*′′,  *J*′′) ← A_1/2_^+^(*v*′,  *J*′) designates the transition between the rovibrational level with vibrational and rotational quantum numbers *v*′ and *J*′ of the *Ω* = 1/2 spin–orbit component of the A^+ 2^Π_*Ω*_ electronic state of MgAr^+^ and the rovibrational level with quantum numbers *v*′′ and *J*′′ of the *Ω* = 1/2 spin–orbit component of the 3dπ_*Ω*_^+^ Rydberg state of MgAr^+^. More generally, we designate the rotational and vibrational quantum numbers of the a state of MgAr with unprimed letters, those of the X^+^, A_*Ω*_^+^ and 3dπ_*Ω*_^+^ of MgAr^+^ using letters with a plus (^+^), a prime (′) and a double-prime (′′) superscript, respectively, and those of the X^2+^ state of MgAr^2+^ with letters with a double-plus (^2+^) superscript. The integer quantum numbers *N*^+^ and *N*^2+^ used to label the rotational levels of the electronic states of Σ symmetry correspond to the standard notation for states following Hund's angular-momentum coupling case (b) and the quantum numbers *J*, *J*′ and *J*′′ used for the rotational levels of electronic states of Π symmetry correspond to the standard notation for states following Hund's angular-momentum coupling case (a), as described, *e.g.*, in ref. 37 and 38, to which we refer for details.

**Table 1:** State labels, term symbols, dissociation asymptotes, and quantum numbers of the rovibronic states of MgAr, MgAr^+^, and MgAr^2+^ studied in the present work

State label	Molecule	Diss. asymptote	Term symbol	Rovibrational quantum numbers
a	MgAr	Mg(3s3p ^3^P) + Ar(^1^S_0_)	^3^Π_0_	*v*, *J*
X^+^	MgAr^+^	Mg^+^(3s) + Ar(^1^S_0_)	^2^Σ^+^	*v* ^+^, *N*^+^
A_*Ω*_^+^	MgAr^+^	Mg^+^(3p) + Ar(^1^S_0_)	^2^Π_*Ω*_	*v*′, *J*′
3dπ_*Ω*_^+^	MgAr^+^	Mg^+^(3d) + Ar(^1^S_0_)	^2^Π_*Ω*_	*v*′′, *J*′′
X^2+^	MgAr^2+^	Mg^2+^(^1^S_0_) + Ar(^1^S_0_)	^1^Σ^+^	*v* ^2+^, *N*^2+^

All results presented below are for ^24^MgAr and its ions, unless stated otherwise.

## 2 Experimental section

### 2.1 Materials and sample preparation

The experimental setup has been presented in ref. 40 and 41. Neutral MgAr in the metastable a(*v* = 0) ground vibrational state was produced by laser ablation of a rod of natural Mg (^24^Mg (79%), ^25^Mg (10%), and ^26^Mg (11%)) in a supersonic expansion of Ar gas. The molecular beam passed through a 3 mm-diameter skimmer located 8 cm downstream of the ablation source before entering the photoexcitation chamber, where it was intersected at right angles by four co-propagating Nd:YAG-pumped dye lasers (25 Hz repetition rate, ∼4 ns pulse duration) that were frequency doubled or tripled in β-barium borate crystals. We refer to these lasers as lasers 1 to 4 below. The laser wavenumbers were calibrated using a commercial wavemeter with a specified accuracy of 0.02 cm^−1^. Photoexcitation took place within an electrode stack used to apply pulsed electric potentials to field ionize high Rydberg states and extract the produced photoelectrons and photoions into a linear time-of-flight (TOF) spectrometer. MgAr^+^ was produced in the lowest vibrational levels (*v*^+^ ≤ 5) of the X^+^ electronic ground state by photoionization of metastable MgAr using laser 1 at *ν̃*_1_ = 39 239 cm^−1^. The particle density of MgAr^+^ in the X^+^(*v*^+^ = 5) level was estimated to be ∼10^4^ cm^−3^ based on the signal intensity in the TOF spectrum, corresponding to ∼200 ions in an interaction volume of ∼0.02 cm^3^ (see also ref. 40). The population of rotational levels in the X^+^ state is well described by a temperature of ∼4 K, as determined from the analysis of the rotational structure of the spectrum of the A_*Ω*_^+^(*v*′) ← X^+^(*v*^+^) transition (see below and ref. 42 and 43).

### 2.2 Photoexcitation sequence

For the photoionization of MgAr^+^ with lasers 2 to 4, we employed the resonant three-photon excitation sequence depicted in Fig. 2. Lasers 2, 3, and 4 were fired simultaneously ∼10 ns after laser 1. Laser 2 was used to pump rovibronic transitions A_1/2_^+^(*v*′ = 1 and 2, *J*′) ← X^+^(*v*^+^ = 5, *N*^+^) at wavenumbers *ν̃*_2_ around 31 253 and 31 513 cm^−1^, respectively. Laser 3 further excited to selected rotational levels of the 3dπ_1/2_^+^(*v*′′ = 2, 3, 4) states at wavenumbers *ν̃*_3_ around 35 597, 35 843, and 35 823 cm^−1^, respectively. The final ionizing transition to the MgAr^2+^ X^2+^ state was induced by laser 4, which was tuned in the range *ν̃*_4_ = 44 400–46 500 cm^−1^. All lasers had the same linear polarization and had a beam diameter of ∼1 mm in the interaction region. The pulse energies of lasers 2, 3, and 4 were typically ∼0.6, ∼0.2, and ∼1 mJ, respectively.

**Fig. 3:**
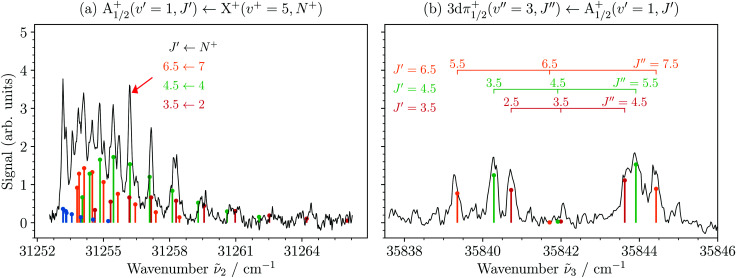
(a) Rotationally resolved spectrum of the A_1/2_^+^(*v*′ = 1,*J*′) ← X^+^(*v*^+^ = 5,*N*^+^) transition of MgAr^+^ recorded by monitoring the Mg^+^ photodissociation yield as a function of the wavenumber *ν̃*_2_ of laser 2.^42,43^ The sticks indicate the calculated positions and intensities of individual rovibronic transitions. The colors blue, orange, green, and red indicate the rotational branches *J*′ − *N*^+^ = −1.5, −0.5, +0.5, and +1.5, respectively. The arrow marks the position in the spectrum used to select the rovibrational levels of the A_1/2_^+^ state with *J*′ = 3.5, 4.5, and 6.5. (b) Rotationally resolved spectrum of the 3dπ_1/2_^+^(*v*′′ = 3,*J*′′) ← A_1/2_^+^(*v*′ = 1, *J*′) transition recorded by monitoring the MgAr^2+^ signal as a function of the wavenumber *ν̃*_3_ of laser 3. The rotational structure reflects the predominantly populated initial rotational levels *J*′ = 3.5, 4.5, and 6.5 of the A_1/2_^+^ state.

The only electronic states of MgAr^+^ that had been characterized prior to this work are the X^+^, A_*Ω*_^+^ and B^+^ states (see Fig. 2).^39,41–46^ The resonant excitation sequence thus necessitated the identification and characterization of a suitable intermediate state between the A_*Ω*_^+^ and the X^2+^ states. In a broad search in the region near the Mg^+^(3d) + Ar(^1^S_0_) dissociation asymptote, we identified the lowest vibrational levels of the 3dπ_1/2_^+^ state to be ideal intermediate states because of their long lifetimes (>50 ps) and large Franck–Condon factors.^47^Fig. 3(a) shows the rotationally resolved spectrum of the A_1/2_^+^(*v*′ = 1, *J*′) ← X^+^(*v*^+^ = 5,*N*^+^) transition recorded by monitoring the yield of Mg^+^ photodissociation product as a function of the wavenumber *ν̃*_2_ of laser 2.^42,43^ The sticks indicate the positions and relative intensities of individual rovibronic transitions, calculated for a rotational temperature of 4 K using standard expressions^37,38^ for transitions between rovibrational levels of ^2^Σ^+^ and ^2^Π_1/2_ states. The red arrow corresponds to the position of laser 2 which selects the *J*′ = 3.5, 4.5, and 6.5 rotational levels from which the spectrum of the 3dπ_1/2_^+^(*v*′′ = 3, *J*′′) ← A_1/2_^+^(*v*′ = 1, *J*′) transition depicted in Fig. 3(b) was measured. This spectrum was recorded by scanning laser 3 and setting laser 4 to 46 290 cm^−1^, while monitoring the MgAr^2+^ signal. It consists of three branches characterized by *J*′′ − *J*′ = 0, ±1 and the assignments are grouped and colored according to the selected A_1/2_^+^ rotational levels.

The band origins 
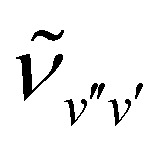
 and the rotational constants 
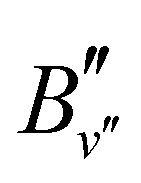
 of the 3dπ_1/2_^+^ levels were determined from the rotational line positions using the formula for the transition energy,^37^1
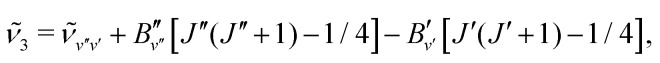
in a least-squares fit. In eqn (1), 
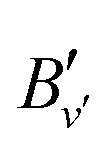
 denotes the rotational constant of the A_1/2_^+^(*v*′) state determined in ref. 43. The results relevant for the present article are summarized in Table 2, where the assignment of the vibrational quantum numbers *v*′′ is based on a standard isotopic-shift analysis.^47,48^ The relative intensities were calculated using well-known expressions for rotational line intensities.^37,38^ In order to avoid power broadening and obtain well-resolved spectra, the pulse energies of lasers 2 and 3 had to be reduced by factors between 5 and 10, compared to the numbers given above, which were required to record the spectra of MgAr^2+^ from the selected 3dπ_1/2_^+^(*v*′′, *J*′′) levels with a sufficient signal-to-noise ratio.

**Table 2:** Observed bands of the transitions from the A_1/2_^+^(*v*′) to the 3dπ_1/2_^+^(*v*′′) states of MgAr^+^ and corresponding band origins and rotational constants 
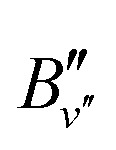
. All values are in units of cm^−1^ and the numbers in parentheses represent one standard deviation in the unit of the last digit

*v*′′	*v*′	**_3_	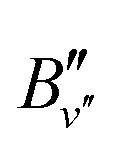
2	1	35596.20(10)	0.182(3)
3	1	35842.13(10)	0.1813(13)
4	2	35823.01(10)	0.1778(22)

### 2.3 PFI-ZEKE-PE and MATI spectroscopy

Pulsed-field-ionization zero-kinetic-energy photoelectron (PFI-ZEKE-PE) spectroscopy^29^ and mass-analyzed threshold ionization (MATI) spectroscopy^30^ are high-resolution variants of threshold photoelectron spectroscopy. Spectra are recorded by monitoring the pulsed-electric-field-ionization yield of very high Rydberg states (principal quantum number *n* > 100) located just below the ionization thresholds as a function of the frequency of a tunable radiation source. After correction for the shifts of the ionization thresholds induced by the electric fields, the positions of the lines observed in the spectra correspond to energy differences between the levels of the ionized molecule (charge *Z*) and its precursor (charge *Z* − 1).

The shift Δ*E*_I_ of the ionization thresholds induced by a single pulsed field of strength *F* is given in good approximation by^49–51^2
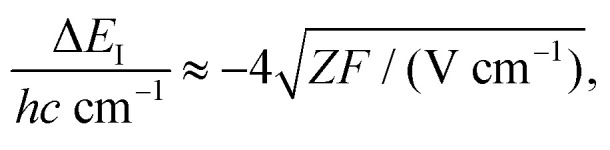
which also gives the expected spectral resolution. To record high-resolution spectra, sequences of several pulsed fields of increasing strength *F*_*i*_ are used (*i* is the pulse index).^52^ In first approximation, the widths *Γ*_*i*_ of the lines obtained by monitoring the field-ionization signal generated by the *i*-th pulse of the sequence are^51^3
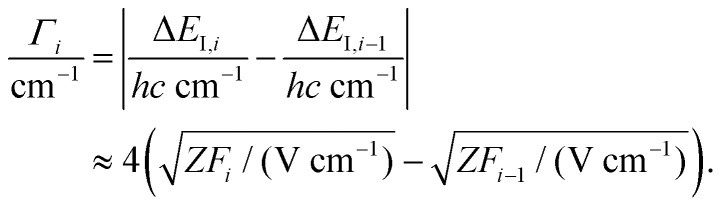
The range of Rydberg states contributing to the PFI signal can be estimated directly from eqn (2) or (3) considering that the energy of a Rydberg state with respect to the ionization threshold is *E* = −*hcZ*^2^*R*/n*^2^, where *R* is Rydberg's constant and n* the effective principal quantum number (see ref. 50 for details).

The main difference between PFI-ZEKE-PE and MATI spectroscopy is that with the former method one detects the electrons generated by the pulsed field ionization whereas in the latter one detects the ions, which offers the advantage of mass selectivity. This advantage was crucial in the present work to separately record the spectra of ^24^MgAr^2+^ and ^26^MgAr^2+^ and determine the absolute assignment of the vibrational levels of MgAr^2+^ from the isotopic shifts. However, the advantage comes at the cost of a reduced resolution because larger electric fields are typically required to distinguish the much heavier ions generated by the successive pulses through their times of flight.

To record the PFI-ZEKE-PE spectra of MgAr^+^ (*Z* = 2) we used a four-pulse sequence (typically *F*_1_ = +0.34 V cm^−1^, *F*_2_ = −0.52 V cm^−1^, *F*_3_ = −1.12 V cm^−1^, and *F*_4_ = −1.72 V cm^−1^) and, for each laser scan, we recorded the three spectra corresponding to *F*_2_, *F*_3_, and *F*_4_ simultaneously. We recorded the MATI spectra with optimized pulse sequences, *e.g.*, *F*_1_ = −0.86 V cm^−1^, *F*_2_ = −1.72 V cm^−1^, and *F*_3_ = +172.4 V cm^−1^, the spectra obtained from *F*_2_ being the high-resolution ones. Because of the small numbers of state-selected MgAr^+^ ions (∼200 per laser shot), the pulsed-field-ionization signal was very weak (less than 1 count per laser shot) and the spectra had to be measured several times and averaged to improve the signal-to-noise ratio.

## 3 Results and discussion

We have measured the spectra of the X^2+^(*v*^2+^ = 0–8) ← 3dπ_1/2_^+^(*v*′′ = 2–4) photoionizing transitions. Our measurements include an overview photoionization spectrum in the vicinity of the *v*^2+^ = 0–2 vibrational levels of the X^2+^ state, PFI-ZEKE-PE spectra of transitions to the X^2+^(*v*^2+^ = 0, 1) levels, and MATI spectra of transitions to the X^2+^(*v*^2+^ = 0–8) levels.

**Fig. 4:**
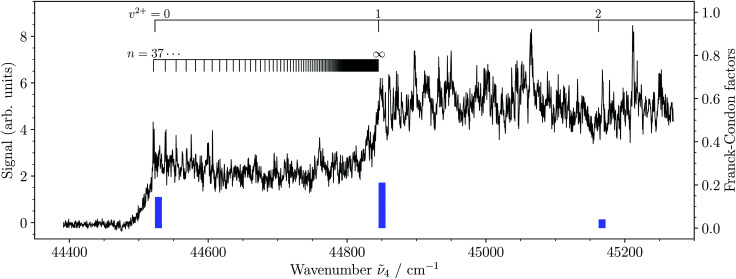
Photoionization spectrum of MgAr^+^ recorded from the intermediate 3dπ_1/2_^+^(*v*′′ = 2) level. The two intensity steps correspond to the first two ionization thresholds as indicated along the upper assignment bar. The lower assignment bar indicates the position of Rydberg states converging to the MgAr^2+^ X^2+^(*v*^2+^ = 1) threshold, several of which are observed as autoionizing resonances just above the X^2+^(*v*^2+^ = 0) level. The blue vertical bars represent the Franck–Condon factors (right vertical axis) calculated with the potential-energy functions of the 3dπ_1/2_^+^ and X^2+^ states. See the text for details.

Fig. 4 depicts the photoionization spectrum of MgAr^+^ from its 3dπ_1/2_^+^(*v*′′ = 2) state to the region of the first three vibrational levels of the ground state of MgAr^2+^. The spectrum shows two distinct steps that indicate the ionization thresholds associated with the *v*^2+^ = 0, 1 levels of the X^2+^ state. Several sharp resonances are also present in the spectrum, which we attribute to autoionizing Rydberg states of MgAr^+^. Just above the ionization threshold associated with the X^2+^(*v*^2+^ = 0) state, we could assign several of these resonances to Rydberg states of MgAr^+^ with principal quantum numbers *n* = 37–40 belonging to series converging to the X^2+^(*v*^2+^ = 1) level of MgAr^2+^, as indicated along the lower assignment bar. The blue bars represent the Franck–Condon factors of the X^2+^(*v*^2+^ = 0–2) ← 3dπ_1/2_^+^(*v*′′ = 2) transitions calculated using the potential-energy functions shown in Fig. 2. The very weak Franck–Condon factor to the X^2+^(*v*^2+^ = 2) state explains why no step could be detected in the photoionization spectrum at the *v*^2+^ = 2 ionization threshold.

**Fig. 5:**
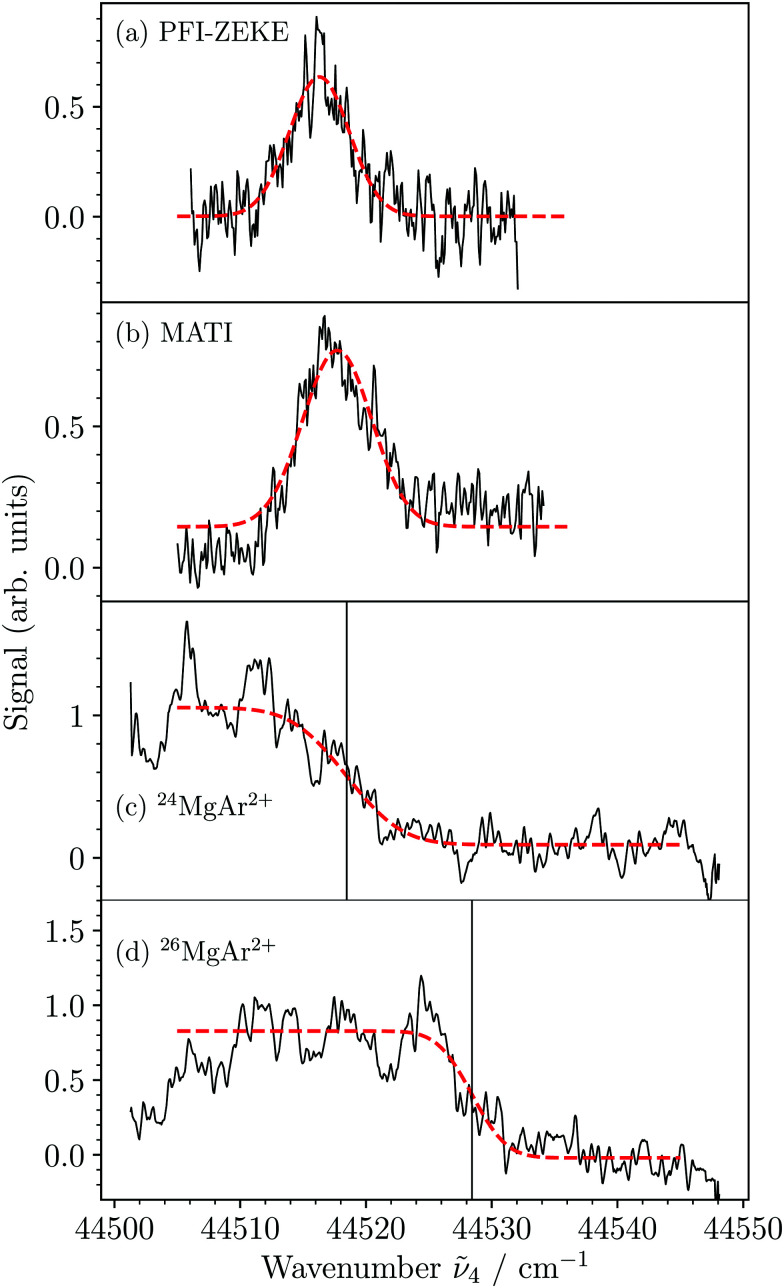
Threshold ionization spectra recorded in the vicinity of the X^2+^(*v*^2+^ = 0) ionization threshold from the intermediate 3dπ_1/2_^+^(*v*′′ = 2) level. (a) PFI-ZEKE-PE spectrum generated by the last pulse of the multipulse sequence +0.34, −0.52, −1.12, and −1.72 V cm^−1^. (b) MATI spectrum generated by the second pulse of the multipulse sequence −0.86, −1.72, and +172.4 V cm^−1^. (c and d) MATI spectra of ^24^MgAr^2+^ and ^26^MgAr^2+^ using the two-pulse sequence −0.86, +172.4 V cm^−1^. The high-energy edges of the spectra were fitted using the error function. The vertical lines designate the corresponding inflection points and their separation corresponds to the isotopic shift of the ionization threshold used for the absolute assignment of the vibrational quantum number.

High-resolution PFI-ZEKE-PE and MATI spectra of the X^2+^(*v*^2+^ = 0) ← 3dπ_1/2_^+^(*v*′′ = 2) transition are presented in Fig. 5(a) and (b). The red dashed lines depict least-squares fits of Gaussian functions to determine the line positions. The slightly asymmetric base line in Fig. 5(b) is caused by an incomplete separation in the TOF spectrum of the MgAr^2+^ signals that were produced by the different field-ionization pulses. Fig. 5(c) and (d) show the corresponding MATI spectra of ^24^MgAr^+^ and ^26^MgAr^+^, respectively, recorded using the two-pulse sequence −0.86, +172.4 V cm^−1^. We determined the isotopic shift (Δ*ν̃*_4_ = 9.7 cm^−1^) of the transition by fitting the error function^53^ to the high-energy edges of the spectra and extracting the difference in the inflection points shown as black vertical lines. From the isotopic shift of the 3dπ_1/2_^+^(*v*′′ = 2) level (*Δ*_3dπ_ = 15.1 cm^−1^, see ref. 47) we determined the isotopic shift of the X^2+^(*v*^2+^ = 0) level to be *Δ*_X^2+^(0)_ = *Δ*_3dπ_ − Δ*ν̃*_4_ = 5.4(20) cm^−1^. A similar measurement of the isotopic shift of the X^2+^(*v*^2+^ = 1) vibrational threshold yielded *Δ*_X^2+^(1)_ = 10.9(20) cm^−1^. These results, together with the vibrational constants determined for ^24^MgAr^2+^ (see below), were used to unambiguously establish the vibrational assignment in a standard isotopic shift analysis.^48^ The fall of the MATI signal below the ionization threshold (Fig. 5(c)) is twice as broad for ^24^MgAr^+^ as it is for ^26^MgAr^+^ (Fig. 5(d)). This difference has its origin in a different initial population of rotational states caused by the slightly different rotational constants and the multiphoton excitation.

**Fig. 6:**
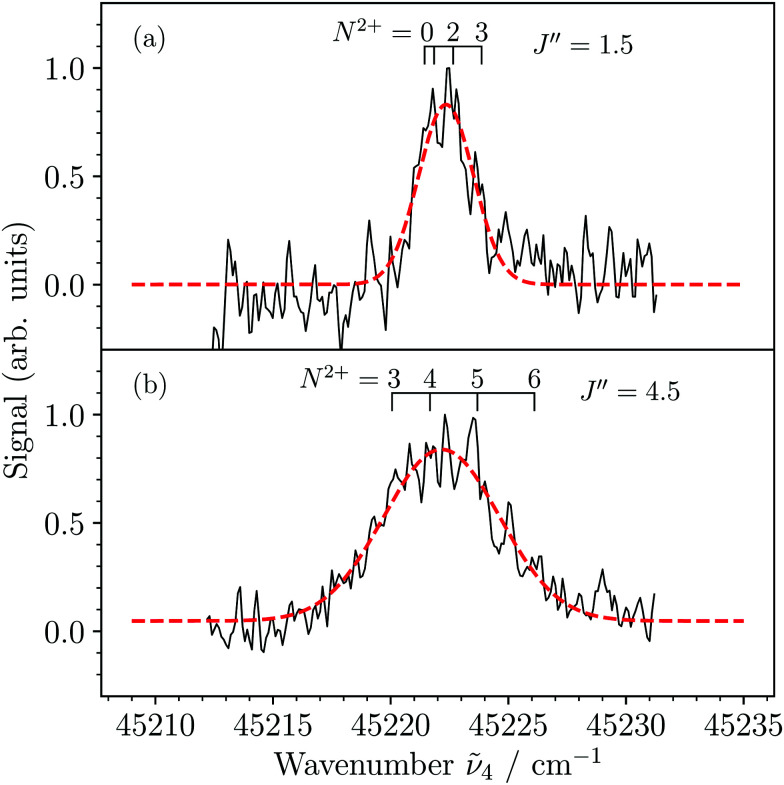
MATI spectra of the X^2+^(*v*^2+^ = 3,*N*^2+^) ← 3dπ_1/2_^+^(*v*′′ = 3,*J*′′) photoionizing transition generated by the third pulse of the pulse sequence +0.26, −1.12, −1.72, and +172.4 V cm^−1^ recorded with predominant population of the *J*′′ = 1.5 (a) and 4.5 (b) rotational levels in the intermediate 3dπ_1/2_^+^(*v*′′ = 3) state. The assignment bars indicate transitions to the rotational levels of MgAr^2+^ assuming dominant branches with *N*^2+^ − *J*′′ = −1.5,…,+1.5.

Fig. 6(a) and (b) show MATI spectra of the X^2+^(*v*^2+^ = 3) ← 3dπ_1/2_^+^(*v*′′ = 3) transition recorded using the sequence of field-ionization pulses of +0.26, −1.12, −1.72, and +172.4 V cm^−1^ and collecting the ionization signal from the −1.72 V cm^−1^ pulse. Although the resolution was not sufficient to resolve the rotational structure in the spectra, we could observe a broadening of the rotational contour when selecting different rotational levels of the 3dπ_1/2_^+^(*v*′′ = 3) state, *i.e.*, *J*′′ = 1.5 (Fig. 6(a)) and *J*′′ = 4.5 (Fig. 6(b)). This effect results from the increasing spread of the rotational transitions at increasing *J*′′ values, as illustrated by the assignment bars, which indicate the expected dominant transitions to rotational levels of the X^2+^(*v*^2+^ = 3) state corresponding to branches with *N*^2+^ − *J*′′ = −1.5,…,+1.5. From simulations (not shown) of the rotational contour using the rotational constants *B*_3_′′ = 0.1813 cm^−1^ (see Table 2) and *B*_3_^2+^ = 0.2017 cm^−1^ (from ref. 36) of the initial and final states, respectively, we estimated the experimental resolution (full width at half maximum of a single line) to be ∼2 cm^−1^. The linewidth estimated from the field-ionization pulse is ∼1.5 cm^−1^ (see eqn (3)) and we attribute this slight discrepancy to power broadening induced by the lasers.

The results of our measurements of the vibrational structure of the X^2+^ ground state of MgAr^2+^ are summarized in Table 3, where all values are corrected for the field-induced shifts of the ionization thresholds. The specified uncertainties are all ≲3 cm^−1^ and represent an improvement in measurement accuracy of approximately two orders of magnitude compared to previous studies of the structure of DIDIs using double-photoionization coincidence methods (see ref. 11 and references therein). The uncertainties of the vibrational term values 
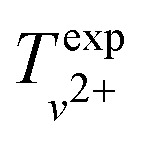
 are smaller than the uncertainties of the transitions because of the cancellation of systematic errors in the determination of the field-induced shifts of the ionization thresholds and in the frequency calibration. From these results, we determined the harmonic and first anharmonic vibrational constants of the X^2+^ state to be *ω*_e_ = 327.02(11) cm^−1^ and *ω*_e_*x*_e_ = 2.477(15) cm^−1^, respectively, which are very close to the values of *ω*_e_ = 328.2 cm^−1^ and *ω*_e_*x*_e_ = 2.55 cm^−1^ calculated *ab initio* by Gardner *et al.*^36^ Using the wavenumbers of the A_1/2_^+^(*v′* = 1) ← X^+^(*v*^+^ = 0) (31704.6 cm^−1^, see ref. 41 and 43) and the 3dπ_1/2_^+^(*v*′′ = 2) ← A_1/2_^+^(*v′* = 1) (35596.2 cm^−1^, see Table 2) band origins, we further determined the adiabatic ionization energy of MgAr^+^ to be *E*_I_(X^+^)/(*hc*) = 111 824(4) cm^−1^, which, to our knowledge, is the most accurate value for the ionization threshold of a molecular cation obtained to date.

**Table 3:** Observed bands in the PFI-ZEKE-PE and MATI spectra of the X^2+^(*v*^2+^) ← 3dπ_1/2_^+^(*v*′′) photoionizing transitions and corresponding band origins and vibrational term values corrected for the field-induced shifts of the ionization thresholds (all values are in units of cm^−1^)

*v* ^2+^	*v*′′	**_4_	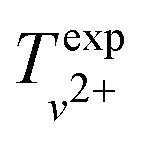 a	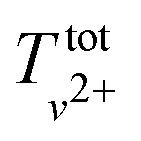 b	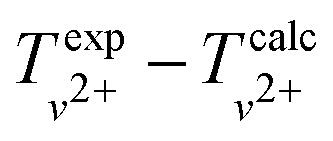 c
0	2	44 523(3)	0.0(10)	111 824(4)	0.8
1	2	44 845(3)	322.0(10)	112 146(3)	0.1
2	3	44916.4(19)	639.0(10)	112463.0(24)	−0.4
3	3	45228.6(19)	951.3(10)	112 775(3)	−0.6
4	4	45296.3(20)	1259.3(10)	113 083(3)	0.1
5	3	45838.0(18)	1560.7(10)	113384.7(24)	−0.6
6	4	45894.6(24)	1857.6(10)	113 682(3)	−0.7
7	4	46 188(3)	2151.0(10)	113 975(3)	0.8
8	4	46474.4(19)	2437.5(10)	114261.5(24)	0.6

a Vibrational term values with respect to the X^2+^(*v*^2+^ = 0) level.

b Vibrational term values with respect to the X^+^(*v*^+^ = 0) level.

c Vibrational term values calculated using the potential function given in eqn (6) and the parameters listed in Table 4.

We determined the dissociation threshold *D*_0_ of the X^2+^ state *via* the thermodynamic cycle4
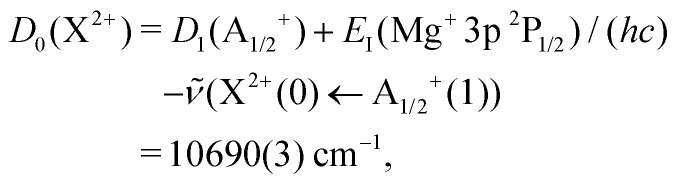
using the known values of the dissociation threshold of the A_1/2_^+^(*v*′ = 1) state^43^ (*D*_1_(A_1/2_^+^) = 5210.7 cm^−1^) and of the ionization energy of the Mg^+^ 3p ^2^P_1/2_ state^54^ (*E*_I_(Mg^+^ 3p ^2^P_1/2_)/(*hc*) = 85598.33 cm^−1^) as well as the wavenumber of the X^2+^(*v*^2+^ = 0) ← A_1/2_^+^(*v*′ = 1) transition, calculated from Tables 2 and 3. The value we obtained for *D*_0_(X^2+^) lies within ∼40 cm^−1^ of the theoretical value of 10730.5 cm^−1^ reported by Gardner *et al.*^36^

To provide an accurate description of the bond in the MgAr^2+^ ground state we used a model potential of the form

5 *V*(*R*) = *V*_Morse_(*R*) + *V*_lr_(*R*)

**Fig. 7:**
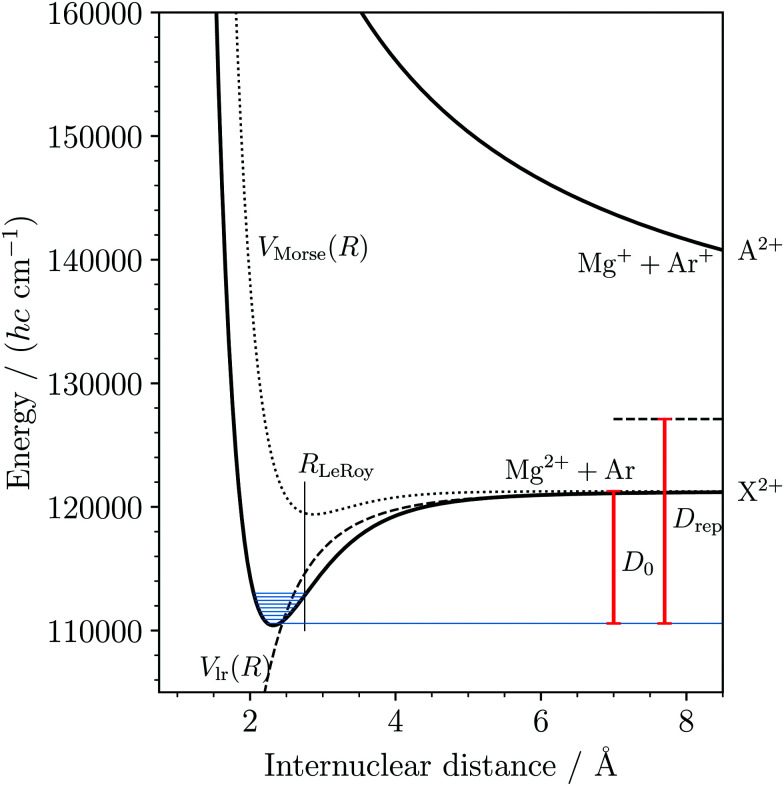
Potential-energy function for the lowest two (X^2+^ and A^2+^) electronic states of MgAr^2+^. The potential-energy function corresponding to the atomic Mg^2+^ + Ar limit was determined by fitting the potential parameters in eqn (6) to match the measured vibrational levels (shown in blue) and the dissociation threshold (*D*_0_). The dashed and dotted lines show the long-range and Morse-type contribution to the potential energy, respectively. The repulsive potential-energy function corresponding to the atomic Mg^+^ + Ar^+^ limit (horizontal dashed line at position *D*_rep_ above the ground rovibronic level) is approximated by a pure Coulomb potential.

6
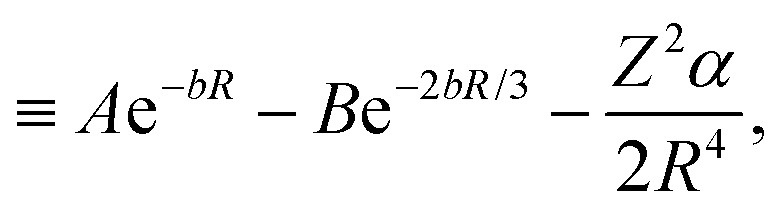
where *A*, *B*, and *b* are adjustable parameters, *Z* = 2, and *α* is set to the known value of the polarizability volume of Ar.^55^ In eqn (6), the first two terms correspond to a generalized Morse potential-energy function and the last term describes the charge-induced-dipole long-range (lr) interaction between Mg^2+^ and Ar. Several authors have used similar model potential functions to describe singly-charged diatomic molecular ions dominated by electrostatic interactions (see, *e.g.*, ref. 56–58 and references therein). We optimized the parameters *B* and *b* in a least-squares fit so as to reproduce our experimental data and fixed the value of *A* corresponding to the *ab initio* value of the equilibrium internuclear distance *R*_e_ = 2.318 Å reported in ref. 36. The level energies were calculated from the potential-energy function by solving the nuclear Schrödinger equation, as described in ref. 41. The values for *A*, *B*, and *b* are listed in Table 4 and the potential is depicted in Fig. 7, together with the contributions from the Morse (dotted line) and the long-range (dashed line) energy functions. The dissociation threshold of 10 690(3) cm^−1^ (127.88(4) kJ mol^−1^) appears surprisingly large at first sight given the rare-gas electron configurations of Ar and Mg^2+^. The attractive part of the potential is dominated by the long-range term, with a minor contribution from the Morse term, which highlights the electrostatic nature of the bond. However, considering that both Mg^2+^ and Ar are chemically hard species, we expected the Morse term to be even less significant. We attribute this weak “chemical” contribution to the binding energy to a charge-transfer interaction with the repulsive MgAr^2+^ A^2+^ state (see also the discussions in ref. 36 for charged alkaline-earth-metal–rare-gas DIDIs and in ref. 59 for the isoelectronic alkali-metal–rare-gas cations). The potential-energy function of the A^2+^ state depicted in Fig. 7 simply corresponds to a repulsive Coulomb potential, which correlates with the dissociation asymptote Mg^+^(3s) + Ar^+^(^2^P_3/2_). This asymptote lies 5842 cm^−1^ above the dissociation limit Mg^2+^ + Ar, corresponding to the difference between the known values of the ionization energies of Ar and Mg^+^.^54^ The strong Pauli repulsion between the full-shell constituents Mg^2+^ and Ar dominates the potential energy at distances below the LeRoy radius^60^*R*_LeRoy_ (vertical line in Fig. 7), where the electron clouds of these atoms start to overlap. *R*_LeRoy_ was calculated in the Hartree–Fock approximation using the program described in ref. 61. The bond of MgAr^2+^ is thus characterized by the interplay of a strongly attractive electrostatic interaction at long range, a weak charge-transfer contribution, and the Pauli repulsion at short range.

**Table 4:** Molecular constants for the MgAr^2+^ X^2+^ state and optimized parameters for the potential-energy function given in eqn (6). The values are in units of cm^−1^ unless indicated otherwise

*ω*_e_	327.02(11)
*ω*_e_ *x*_e_	2.477(15)
*D*_0_(X^2+^)	10 690(3)
*E*_I_(X^+^)/(*hc*)	111 824(4)

*A*	71.30016 *E*_h_
*B*	6.644272 *E*_h_
*b*	1.534148 *a*_0_^−1^
*α*	11.077 *a*_0_^3^ a

a From ref. 55.

## 4 Conclusions and outlook

In this article, we have reported the first spectroscopic characterization of a thermodynamically stable DIDI. We measured the photoionizing transitions to the first nine vibrational levels (*v*^2+^ = 0–8) of the MgAr^2+^ X^2+^ ground state using the techniques of PFI-ZEKE-PE and MATI spectroscopy and were able to observe the effect of the rotational structure on the spectra as well as to determine the isotopic shifts of the X^2+^(*v*^2+^ = 0, 1) levels of ^24^MgAr^2+^ and ^26^MgAr^2+^. From our measurements we could determine accurate values for the ionization energy of MgAr^+^ and the dissociation energy of the ground state of MgAr^2+^. We also derived a potential-energy function that accurately describes the interaction between Mg^2+^ and Ar and unravels the binding mechanisms. The bond is dominated by electrostatic interactions. The double charge of the Mg^2+^ (*Z* = 2) constituent leads to a dissociation energy that is comparable to that of a covalent bond. This behavior follows from the *Z*^2^ (= 4) dependence of the charge-induced-dipole interaction (see eqn (6)). Compared to the singly-charged isoelectronic species (*Z*^2^ = 1) an increase in binding energy of much more than a factor of four is expected because of the reduction of the internuclear separation when increasing *Z*. From eqn (6) and Fig. 7 one would expect the relation7
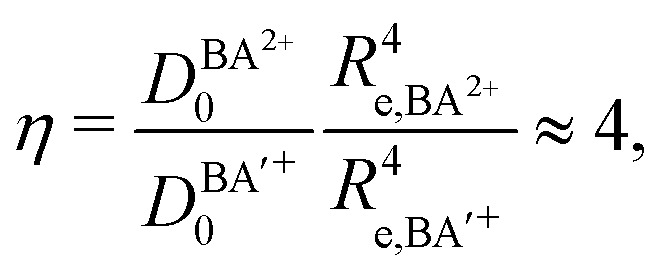
to hold in good approximation for thermodynamically stable DIDIs BA^2+^. In the case of MgAr^2+^ and the isoelectronic singly charged NaAr^+^ one finds *η* = 4.07 using 
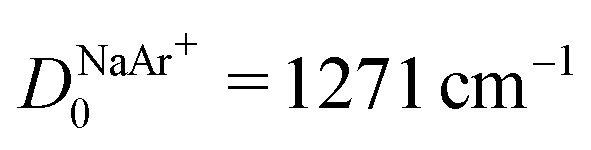
 and *R*_e,NaAr^+^_ = 2.78 Å from ref. 62.

25 years have elapsed since Falcinelli *et al.*^14^ reported their extensive list of thermodynamically stable DIDIs. In this time, progress in the characterization of their structure and dynamics has been exclusively theoretical (see, *e.g.*, ref. 9, 20 and 36 and references therein). This lack of spectroscopic data may appear surprising at first sight but is explainable by experimental challenges. The spectroscopic methods used to study DIDIs, emission^21,26^ and photofragment^22–25,27^ spectroscopy of the doubly charged systems and single-photon or electron-impact double ionization spectroscopy of the neutral parents,^11^ are all particularly challenging for thermodynamically stable DIDIs. The first electronically excited state in these DIDIs is typically repulsive in the Franck–Condon region of the stable vibrational levels (see Fig. 1(c) and (d)) so that electronic spectra are structureless (continuous). Rotational and vibrational absorption or emission spectra in the electronic ground state are in principle observable, but their detection is severely complicated by the very low densities of BA^2+^ molecules that can be generated in the gas phase. The parent neutral molecules BA are highly reactive and must be generated *in situ*, which leads to insufficient concentrations for the detection of single-photon double-ionization coincidence events. In the future, we believe that non-destructive measurements of absorption processes of individual DIDIs in ion traps will offer an attractive route to obtain high-resolution rotational and vibrational spectra of these systems.^63^

In this article, we have demonstrated the successful use of high-resolution photoelectron spectroscopy of the singly-charged parent ion BA^+^ as a powerful method to study DIDIs. The advantages are obvious: (i) the measurement of high-resolution photoelectron spectra does not require high-density samples (here 5000 ions per second at 10^4^ cm^−3^ density) and is ideally suited for the study of charged species, as is well known for anions (see, *e.g.*, ref. 64 and references therein), which can be ionized with commercial VIS and UV lasers. So far its application to cations has been hampered by their high ionization energies. (ii) In the case of thermodynamically stable DIDIs, the ionization energy of BA^+^ is comparable to that of B^+^ (*E*_I_/(*hc*) = 121267.64 cm^−1^ in the case of Mg^+^ ^54^), which is low as explained in Section 1. The metallic nature of B further guarantees that B^+^ and therefore also BA^+^ have low-lying electronic states that can be used as intermediate states to efficiently ionize BA^+^ in resonant multiphoton processes. (iii) The parent cation BA^+^ can be produced in selected rovibrational levels by photoexcitation from the ground state of BA using threshold ionization techniques (see ref. 30 and 65), which can be exploited to access a broad range of vibrational levels of BA^2+^.

Photoelectron spectroscopy of singly charged cations can also be used to study metastable DIDIs. The requirements for the efficient photoionization of BA^+^, however, are more stringent because the energies are higher in this case and adequate multiphoton ionization sequences may be more difficult to find. Nevertheless, the list of metastable DIDIs presented in the review of Sabzyan *et al.*^9^ makes one optimistic that several of them can be studied with the method presented here. Studies of He_2_^2+^, O_2_^2+^, and CO^2+^ would be of particular importance for fundamental reasons and also for applications in atmospheric chemistry and astrophysics. In the future, we expect that progress in the development of powerful narrow-band VUV radiation at free-electron laser facilities will offer the possibility of efficiently ionizing molecular cations in single-photon processes and recording their high-resolution single-photon photoelectron spectra.

The experimental method presented here to study molecular dications is not restricted to DIDIs, but can of course be equally well applied to polyatomic systems.

## Conflicts of interest

There are no conflicts to declare.
